# Metabolic Health in Relation to Body Size: Changes in Prevalence over Time between 1997-99 and 2008-11 in Germany

**DOI:** 10.1371/journal.pone.0167159

**Published:** 2016-11-23

**Authors:** Julia Truthmann, Gert B. M. Mensink, Anja Bosy-Westphal, Christa Scheidt-Nave, Anja Schienkiewitz

**Affiliations:** 1 Department of Epidemiology and Health Monitoring, Robert Koch Institute, Berlin, Germany; 2 DZHK (German Centre for Cardiovascular Research), partner site Berlin, Germany; 3 Institute of Nutritional Medicine, University of Hohenheim, Stuttgart, Germany; Children's National Health System, UNITED STATES

## Abstract

**Objective:**

The study examined potential changes in the proportion of metabolic health according to body size categories over time and across strata of sex and age, varying definitions of metabolic health.

**Methods:**

We analysed data from national health interview and examination surveys 1997–99 and 2008–11 for adults aged 18–79 years (GNHIES98: N = 6,565; DEGS1: 6,860). Metabolic health as defined by ATPIII criteria was examined across body mass index categories. The Plourde and Karelis criteria were applied in relation to abdominal obesity.

**Results:**

Proportions of adults with metabolic health by body size categories were largely stable over time, except for an increasing proportion of metabolically healthy persons with pre-obesity and metabolically healthy women without abdominal obesity. In both surveys proportions of adults meeting ATPIII criteria ranged from approximately 30% among men and women with obesity, to about two thirds of those with pre-obesity to about 93% among those with normal weight. According to Plourde and Karelis criteria proportions ranged from almost 30% among men and women without abdominal obesity to less than 10% among those with abdominal obesity. Proportions were consistently higher among younger than older age groups and less consistently higher among women than men.

**Conclusions:**

Proportions of adults with metabolic health by body size categories were largely stable over time, except for an increasing proportion of metabolically healthy women without abdominal obesity. There is no evidence that metabolic health among adults with obesity increased in Germany over a period of ten years.

## Introduction

Worldwide obesity increased from 1975 to 2014 [1] and the prevalence of obesity in Germany also increased over the first decade of the 21^st^ century, especially among young adults [2]. While obesity is closely related to clustering of metabolic risk factors and increased morbidity and mortality risk [3], a substantial proportion of persons with obesity do not fulfil commonly applied cut-off criteria to define adverse metabolic risk profiles in cross-sectional epidemiological studies. This has fuelled discussion on whether there may be a metabolically more benign or metabolically healthy (MH) subgroup of persons with obesity with a better prognosis than metabolically unhealthy (MU) persons [4]. Vice versa, normal weight persons already express cardio-metabolic risk factor clustering, and this normal weight MU subgroup may be at high risk of morbidity and mortality in the absence of obesity [5]. Previous studies reported a highly variable proportion of MH people with obesity ranging from 10 to 40% based on varying criteria to define the metabolic syndrome (MetS) and insulin sensitivity [6, 7]. However, there is ongoing controversy regarding the public health relevance of MH in the presence of obesity [8, 9], since it may shift the focus away from the major problem of an increasing prevalence of obesity [10]. Furthermore, recent meta-analyses suggest that MH persons with obesity have an intermediate metabolic health status with increased risk for cardiovascular disease (CVD) [11] and mortality [12] compared to MH normal weight persons, but lower risk compared to MU persons with obesity.

The mechanisms to explain the favourable metabolic profile of MH persons with obesity are still unknown, but seem to be associated with the individual fat distribution [4]. A more favourable fat distribution with more subcutaneous and less visceral adipose tissue could be determined by genetic [13], as well as behavioural and environmental factors [14]. Recent analyses of national health studies in Germany 1997–1999 (GNHIES98) and 2008–11 (DEGS1) demonstrated favourable changes in several cardio-metabolic risk factors [15–18], albeit not in high blood pressure also including blood pressure treatment [19]. In the US [20] and northern Sweden [21] CVD risk factors except diabetes have improved, and population-based data from Denmark demonstrated an upward shift towards higher values of optimal body mass index (BMI) associated with lowest mortality from all and cardiovascular causes [22]. Therefore it remains unclear if the prevalence of MH obesity has changed over time.

Against this background, the present study aimed: (1) to investigate potential changes in the proportion of MH according to body size categories over time using comparable data from the two recent National Health Interview and Examination Surveys for adults in Germany, (2) to examine if the changes were consistent across strata of sex and age.

## Methods

### Study design and population

The National Health Interview and Examination Surveys for adults in Germany 1997–99 (GNHIES98) and 2008–11 (DEGS1) were conducted as part of the German health monitoring system. In a two-stage sampling procedure 180 sample-points (study locations) were selected reflecting the distribution of the population at the regional level. Then age- and sex-stratified random samples of the population 18–79 years were selected from population-registries in every sample point. For DEGS1 the sample consists of 4,193 first-time participants and 3,959 persons, who had already participated in GNHIES98. The concept of both surveys is described in detail elsewhere [23, 24]. The response rate was 61% in GNHIES98, 62% for re-participants in DEGS1 and 42% for first-time participants in DEGS1. Non-participants were asked to fill in a short questionnaire including information on socio-demographic and health-related characteristics. The comparison between responders and non-responders of DEGS1 and between the overall net sample and the resident population of Germany supported high representativeness [25].

The net sample of 7,124 participants for GNHIES98 and 7,115 participants for DEGS1 had participated in the examination part and allows representative cross-sectional analyses for the age range of 18–79 years and time trend analyses. Participants with missing data on the definition of MH and body size category were excluded, resulting in a final study sample of 6,565 for GNHIES98 and 6,860 for DEGS1. Both studies were conducted according to the Federal and State Commissioners for Data Protection guidelines. DEGS1 was approved by the local ethics committee at Charité-Universitätsmedizin Berlin in October 2008 (ethics approval application document number: EA2/047/08). The implementation of the surveys conforms to the principles of the Helsinki Declaration. All participants provided written informed consent prior to survey participation.

### Data collection and study variables

Standardized measures of body weight, height, and waist circumference (WC) were obtained from participants wearing light clothing without shoes (GNHIES98) or underwear without shoes (DEGS1). Body weight was measured using a calibrated electronic scale (SECA, column scale 930) with a precision of 0.1kg. In GNHIES98 body height was measured with a leveling board on the electronic scale and in DEGS1 with a portable stadiometer (Holtain Ltd., UK), both with a precision of 0.1 cm. The body mass index (BMI) was calculated using the formula: BMI [kg/m^2^] = body weight [kg]/body height² [m]. WC was measured at the minimal waist using a flexible, non-stretchable tape. Among participants with obesity (GNHIES98) and participants with no visible waist (DEGS1) WC was measured at the midpoint between the lowest rib and the ileac crest.

Self-reported physician-diagnosed diabetes, hypertension, and dyslipidaemia were obtained in computer-assisted interviews administered by specifically trained study physicians. All medications taken in the seven days prior to the interview were documented by trained medical staff. Medications were coded according to the Anatomical Therapeutic Chemical classification system and categorized as any lipid-lowering (C10), antihypertensive (C02, C03, C07, C08, C09), and antidiabetic medication (A10).

Blood samples were taken at the study centres, processed within one hour, and stored at -40°C. The fasting period was documented for every participant. Fasting instructions changed between surveys. Blood samples were taken over the day in GNHIES98 [23]. In DEGS1, participants were specifically asked to keep an overnight fasting period of at least eight hours for morning appointments and of at least four hours for afternoon appointments, unless they had diagnosed diabetes [24]. Consequently, a significantly higher proportion of study participants had fasting blood samples in DEGS1 compared to GNHIES98 [18]. A fasting period less than eight hours was categorized as non-fasting. Laboratory analyses were conducted at the Robert Koch Institute Central Epidemiological Research Laboratory. In GNHIES98 glycated haemoglobin (HbA1c) was determined by a Diamant high performany liquid chromatography analyser (Bio-Rad, Germany) using reagents from Recipe (Recipe Chemicals and Instruments, Munich) and in DEGS1 by an immunoturbidimetric method (Architect ci8200). Measurements from both methods were traceable to the National Glycohemoglobin Standardization Program reference and the distribution of HBA1c measures among metabolically healthy subgroups of normoglycaemia did not change over time [26]. Serum lipid levels were determined by an enzymatic procedure, based on the cholesterol oxidase-peroxidase 4-aminophenazone method (high density lipoprotein-cholesterol, HDL-C) and the glycerol-3-phosphate oxidase-peroxidase 4-aminophenazone method (GPO-PAP; triglycerides). While the principle of measurement remained the same, the analyser for serum lipids changed during the surveys (GNHIES98: MEGA, Merck, Germany; DEGS1: Architect ci2800, Abbott, Germany). A small impact cannot be excluded, but previous analyses do not suggest substantial measurement error [18].

Blood pressure was measured on the right arm following a standardized protocol and using either a standard mercury sphygmomanometer (GNHIES98) or an automated oscillometric device (Datascope Accutorr Plus, DEGS1). The blood pressure values of GNHIES98 were corrected by a calibration formula validated for DEGS1 [27].

### Definition of metabolic health and body size category

We defined MH using two different established algorithms, which were applicable to our survey data. First, we defined MH by the absence of the MetS based on the National Cholesterol Education Program ATP III criteria [28] as used by van Vliet-Ostaptchouk et al. [29]. According to this algorithm, MH is present among persons fulfilling at least three out of five criteria as listed in Table 1. Use of any antidiabetic, antihypertensive or lipid-lowering medication as well as history of physician-diagnosed diabetes, hypertension and dyslipidaemia is additionally considered and counted as not fulfilling the respective criterion. Secondly, MH was defined using the criteria proposed by Plourde and Karelis (PK criteria) [30]. These provide a considerably more stringent algorithm to define MH, as they include lower thresholds for blood pressure and require fulfilment of all of the four proposed criteria. Because fasting instructions changed between GNHIES98 and DEGS1 we used HbA1c, which is independent of fasting time, instead of serum glucose. Different cut-offs for triglycerides were used among fasting and non-fasting participants as described by van Vliet-Ostaptchouk et al. [29].

**Table 1:** Definition of metabolic health.

	ATPIII criteria	Plourde and Karelis criteria
Waist circumference	< 88 cm (women) or 102 cm (men)	-
HbA1c	< 5.7% [32] and no diagnosis of diabetes and no use of antidiabetic medications	< 5.7% [32] and no diagnosis of diabetes and no use of antidiabetic medications
Blood pressure	< 130/85 mmHg and no diagnosis of hypertension and no use of antihypertensive medication	< 120/80 mmHg and no diagnosis of hypertension and no use of antihypertensive medication
Fasting triglycerides	Fasting triglycerides< 1.7 mmol/l or non-fasting triglycerides< 2.1 mmol/l [29] and no diagnosis of dyslipidaemia and no use of lipid-lowering medication	Fasting triglycerides< 1.7 mmol/l or non-fasting triglycerides< 2.1 mmol/l [29] and no diagnosis of dyslipidaemia and no use of lipid-lowering medication
HDL-C	≥ 1.03 mmol/l (men) or 1.30 mmol/l (women)	≥ 1.03 mmol/l (men) or 1.30 mmol/l (women)
Metabolic health	At least three criteria fulfilled	All criteria fulfilled

Body size categories were defined as BMI categories of normal weight (BMI< 25 kg/m²), pre-obesity (25 kg/m²≤ BMI< 30 kg/m²), and obesity (BMI≥ 30 kg/m²) according to WHO recommendations [31] or WC categories indicating individuals without abdominal obesity (WC< 80 cm among women and WC< 94 cm among men) and those with abdominal obesity (WC≥ 80 cm among women and WC≥ 94 cm among men) [30].

### Analysis

In cross-sectional survey-specific analyses means, percentages and 95% confidence intervals were calculated sex-specifically and for men and women combined. The proportion of persons with MH across BMI categories were assessed using ATPIII criteria, whereas WC categories were applied in relation to the PK criteria of MH. Differences in the proportions of MH by body size categories between the two survey periods were tested using the Rao-Scott chi-square test. In a sensitivity analysis we excluded WC from the definition of MH by ATPIII criteria (two out of four criteria fulfilled) as done in previous studies [29, 32]. The proportion of MH by body size categories in 1997–99 and 2008–11 was also calculated stratified for age (18–44, 45–64, and 65–79 years).

All statistical analyses were performed using survey procedures for complex samples in SAS 9.4 (SAS Institute, Cary, NC). To account for the unequal sampling probabilities, the statistical analyses were weighted. The weighting factor for DEGS1 was calculated in two steps [25]. First, design weights were calculated separately for first-time participants and re-participants. The weighting factor for re-participants was the inverse participation probability in DEGS1 (product of the probability of having participated in GNHIES98 and the probability to participate again in DEGS1). In the second step, both design weights were adjusted to the population structure in Germany on 31.12.2010 with regard to age, sex, educational status, federal state, and type of municipality. Results for GHNIES98 were adjusted to the population structure on 31.12.1997. For analysis of differences in proportions over time, the GNHIES98 results were age-standardized to the population on 31 December 2010. P values were calculated based on two-sided tests. Bonferroni-Holm correction (α = 0.05) was used to reduce the chances of obtaining false-positive results (type 1 errors). With regard to multiple comparisons each p value< 0.001 was considered statistical significant.

## Results

The study characteristics of GNHIES98 and DEGS1 are presented in Table 2. More participants in DEGS1 fasted longer than eight hours before blood sampling. Compared to participants of GNHIES98 those of DEGS1 had a higher prevalence of obesity based on BMI, which was due to increases among men. In contrast, the prevalence of abdominal obesity decreased among men. Participants in DEGS1 showed also changes in a number of cardio-metabolic criteria compared to GNHIES98 participants, including a higher proportion of men with low risk triglyceride levels, high HDL-C levels and a higher proportion of women with very low risk blood pressure and low risk HbA1c levels. In contrast, a lower proportion of men had low risk blood pressure in DEGS1 compared to GNHIES98.

**Table 2:** Characteristics of the study population by survey, National Health Interview and Examination Surveys for Adults in Germany 1997–99 (GNHIES98) and 2008–11 (DEGS1).

	Total			Men			Women		
	GNHIES98 N = 6,565	DEGS1 N = 6,860	P	GNHIES98 N = 3,205	DEGS1 N = 3,298	P	GNHIES98 N = 3,360	DEGS1 N = 3,562	P
Age (years; mean)	45.4 (44.8–46.1)	47.3 (46.8–47.8)	.850	44.4 (43.6–45.2)	46.9 (46.2–47.5)	.860	46.4 (45.7–47.1)	47.8 (47.1–48.4)	.890
Fasting duration≥ 8 hours (%)	26.5 (25.1–28.1)	48.0 (46.5–49.5)	**< .001**	22.9 (20.9–24.9)	50.2 (48.2–52.3)	**< .001**	30.2 (28.2–32.3)	45.7 (43.7–47.7)	**< .001**
**Body size groups**									
Underweight (BMI< 18.5 kg/m², N)	59	91		12	24		47	67	
Normal weight (BMI< 25 kg/m², %)	39.3 (37.5–41.2)	40.2 (38.5–41.8)	**< .001**	32.3 (30.0–34.6)	32.9 (30.9–35.0)	**< .001**	46.3 (44.0–48.6)	47.4 (45.2–49.6)	.110
Pre-obesity (25≤ BMI< 30 kg/m², %)	39.6 (38.2–41.0)	36.4 (35.1–37.7)		48.6 (46.6–50.6)	43.6 (41.6–45.6)		30.7 (29.0–32.4)	29.2 (27.5–31.0)	
Obesity (BMI≥ 30 kg/m², %)	21.1 (19.6–22.7)	23.5 (21.9–25.0)		19.1 (17.6–20.8)	23.5 (21.4–25.7)		23.0 (21.0–25.1)	23.4 (21.5–25.5)	
Abdominal obesity (waist circumference≥ 80/94 cm ^a^, %)	58.6 (56.6–60.5)	55.6 (53.5–57.5)	**< .001**	57.6 (55.2–60.0)	54.2 (51.7–56.6)	**< .001**	59.5 (57.1–61.8)	56.9 (54.4–59.5)	.008
**Cardio-metabolic criteria (%)**									
Waist circumference< 88/102 cm^a^	66.1 (64.3–67.9)	66.1 (64.2–68.0)	.110	70.5 (68.4–72.5)	68.7 (66.3–70.9)	.690	61.7 (59.4–64.0)	63.6 (61.1–66.1)	.025
Low risk HbA1c (< 5.7%)^b^	65.1 (62.7–67.5)	69.8 (66.6–72.9)	**< .001**	62.5 (59.6–65.4)	65.4 (61.6–69.0)	.032	67.7 (65.2–70.1)	74.2 (70.8–77.3)	**< .001**
Very low risk blood pressure (< 120/80 mmHg)^c^	31.6 (29.8–33.5)	32.0 (30.5–33.5)	.013	24.4 (22.4–26.5)	20.5 (18.8–22.3)	.110	38.8 (36.3–41.2)	43.4 (41.3–45.5)	**< .001**
Low risk blood pressure (< 130/85 mmHg)^c^	53.9 (52.0–55.8)	48.7 (47.3–50.1)	.029	51.3 (48.8–53.8)	41.5 (39.4–43.6)	**< .001**	56.5 (54.4–58.6)	55.9 (53.9–57.8)	.170
Low risk triglycerides^d^	59.2 (57.7–60.8)	60.2 (58.8–61.5)	.005	51.9 (49.6–54.2)	54.6 (52.6–56.6)	**< .001**	66.5 (64.4–68.5)	65.7 (63.8–67.6)	.540
High HDL-C (≥ 1.30/1.03 mmol/l)^a^	74.6 (73.0–76.2)	79.5 (77.6–81.3)	**< .001**	72.9 (70.8–74.9)	82.3 (80.0–84.3)	**< .001**	76.4 (74.2–78.4)	76.8 (74.3–79.1)	.750

Due to Bonferroni-Holm correction p values < 0.01 were considered statistically significant (bold).

^a^ women/men.

^b^ and no diagnosis of diabetes and no antidiabetic medication.

^c^ and no diagnosis of hypertension and no antihypertensive medication.

^d^ fasting triglycerides< 1.7 mmol/l (2.1 mmol/l among individuals who fasted less than eight hours) and no diagnosis of dyslipidaemia and no lipid-lowering medication.

From GNHIES98 to DEGS1 proportions of persons with MH were unchanged among all categories of body size (Table 3), except a small increase in MH among persons with pre-obesity (65 to 69%) and among women without abdominal obesity (38 to 43%). In sensitivity analyses excluding WC from the ATPIII criteria no changes in MH were found in all BMI categories between GNHIES98 and DEGS1 (S1 Table). Proportions were substantially and consistently lower applying the more stringent PK criteria as compared to the ATPIII criteria. Proportions of persons with MH were consistently higher among normal weight compared to persons with pre-obesity and obesity, as well as among persons without abdominal obesity compared to participants with abdominal obesity. In both surveys the proportion of men and women combined with obesity meeting ATPIII criteria was almost 30% and the proportion of men and women with MH according to PK criteria was less than 10% among those with abdominal obesity.

**Table 3:** Proportion (%) of metabolically healthy persons by body size categories and survey, National Health Interview and Examination Surveys for Adults in Germany 1997–99 (GNHIES98) and 2008–11 (DEGS1).

	Total			Men			Women		
	GNHIES98 N = 6,565	DEGS1 N = 6,860	P	GNHIES98 N = 3,205	DEGS1 N = 3,298	P	GNHIES98 N = 3,360	DEGS1 N = 3,562	P
**ATPIII criteria**									
Normal weight	92.3 (91.0–93.4)	93.0 (91.6–94.1)	.033	90.5 (88.4–92.3)	91.7 (89.2–93.7)	.081	93.5 (91.8–94.8)	93.9 (92.2–95.2)	.200
Pre-obesity	65.4 (63.1–67.7)	68.6 (66.3–70.9)	**< .001**	66.8 (63.8–69.7)	69.0 (66.0–71.8)	.011	63.2 (60.2–66.1)	68.1 (64.5–71.4)	.004
Obesity	26.3 (22.9–30.0)	32.0 (28.9–35.4)	.002	23.5 (19.2–28.4)	30.6 (26.1–35.4)	.014	28.6 (24.0–33.7)	33.5 (29.5–37.8)	.033
**Plourde and Karelis criteria**									
No abdominal obesity	27.8 (25.6–30.1)	30.9 (28.7–33.3)	.003	18.0 (15.7–20.7)	19.2 (16.6–22.2)	.330	37.8 (34.5–41.2)	43.3 (40.2–46.5)	**< .001**
Abdominal obesity	7.7 (6.7–8.9)	7.6 (6.4–8.9)	.480	3.9 (2.9–5.3)	3.6 (2.6–5.1)	.870	11.4 (9.8–13.2)	11.3 (9.6–13.3)	.550

Due to Bonferroni-Holm correction p values < 0.01 were considered statistically significant (bold).

In age-specific analyses proportions of MH were consistently higher in younger compared to older age groups (Table 4). There was an increase over time in the proportion of persons with MH among 18–44 year-old men with obesity (33 to 52%) and 65–79 year-old women with normal weight (62 to 79%), although the result was not statistically significant according to the Bonferroni-Holm correction.

**Table 4:** Proportion (%) of metabolically healthy^a^ persons by body mass index and survey, National Health Interview and Examination Surveys for Adults in Germany 1997–99 (GNHIES98) and 2008–11 (DEGS1) stratified by age group.

	18–44 years			45–64 years			65–79 years		
	GNHIES98	DEGS1	P	GNHIES98	DEGS1	P	GNHIES98	DEGS1	P
**Men**	N = 1608	N = 1158		N = 1197	N = 1250		N = 400	N = 890	
Normal weight	95.7 (93.4–97.2)	95.8 (92.7–97.6)	.890	81.5 (75.7–86.1)	88.4 (82.8–92.3)	.090	64.7 (51.6–75.9)	72.8 (63.1–80.7)	.270
Pre-obesity	81.9 (78.0–85.2)	83.2 (78.0–87.4)	.420	58.6 (53.4–63.6)	68.7 (64.4–72.7)	.006	42.8 (36.0–49.9)	44.5 (38.5–50.5)	.560
Obesity	32.8 (25.1–41.6)	51.5 (42.8–60.0)	.003	19.2 (14.4–25.1)	26.2 (20.5–32.7)	.120	10.9 (5.9–19.3)	11.5 (6.9–18.6)	.780
**Women**	N = 1624	N = 1260		N = 1245	N = 1422		N = 491	N = 880	
Normal weight	98.3 (96.8–99.1)	98.8 (97.3–99.5)	.390	90.2 (86.4–93.1)	89.6 (85.8–92.4)	.700	61.7 (51.3–71.0)	78.5 (70.7–84.7)	.007
Pre-obesity	89.3 (85.9–92.0)	92.8 (88.6–95.5)	.100	60.4 (55.6–65.1)	69.6 (64.3–74.4)	.036	31.3 (25.0–38.3)	35.1 (28.8–42.0)	.410
Obesity	52.5 (43.2–61.6)	60.6 (51.2–69.3)	.230	23.1 (18.6–28.3)	32.2 (26.9–38.0)	.021	11.9 (6.7–20.5)	15.3 (11.0–20.9)	.410

Due to Bonferroni-Holm correction p values < 0.01 were considered statistically significant (bold).

^a^ defined by ATPIII criteria.

## Discussion

The proportion of MH persons with normal weight, obesity and abdominal obesity remained unchanged between GNHIES98 and DEGS1 based on the original ATPIII and the PK criteria. In contrast, there was a slight increase in the proportion of MH among persons with pre-obesity and among women without abdominal obesity. Using the ATPIII criteria 93% of men and women combined with normal weight, 69% with pre-obesity (65% in GNHIES98) and 32% with obesity were MH in DEGS1. Using the more stringent PK criteria only 31% of persons without abdominal obesity (28% in GNHIES98) and 8% of persons with abdominal obesity were MH in DEGS1. In accordance with previous studies [29, 33], a higher proportion of MH was found among women compared to men.

The increasing proportion of MH among persons with pre-obesity and women with abdominal obesity are in line with the favourable changes in health-related behaviour in the German population between GNHIES98 and DEGS1. During this period, the prevalence of sports activity [15] and fruit intake increased [17], whereas the prevalence of smoking [16] and mean serum lipid levels decreased [18]. An improvement in MH among all anthropometric categories was found between 1986 and 2009 in northern Sweden (MONICA study; Multinational MONItoring of trends and determinants in CArdiovascular disease) [34]. In this study population, improved MH defined by normal blood pressure (< 140/90 mmHg), normal cholesterol (< 5.0 mmol/l) and no known diabetes. However, increased prevalence of MH was not consistently found in all sex and weight strata over the entire study period.

We found a higher proportion of MH among younger age groups. Aging is associated with the conversion from the MH to the MU subgroup as seen in longitudinal studies indicating that not all MH individuals remain healthy over time [35, 36]. This is partly due to the fact that weight gain and changes to a less favourable behavioural pattern all become more likely with increasing age [35]. The duration of overweight also plays an important role in the progression of CVD risk factors [37].

Multiple comparisons enhance the chance to obtain a false-positive finding. To reduce this chance we corrected for multiple testing using the Bonferroni-Holm method. On the other hand, the correction for multiple testing increases the type II error (false-negative finding) [38]. Although the hypothesis of increasing MH among young men with obesity was rejected due to the Bonferroni-Holm correction, the results are in line with previous findings regarding the transition of the MH group. Previous studies do not demonstrate a greater improvement in health-behaviour among young men than among men at older ages [15–17]. This suggests that the increase in MH among 18–44 year-old men with obesity are due to the increased prevalence of obesity among this age group [2]. The duration of obesity has great impact on the metabolic profile and the increased proportion of MH among this group may be attenuated after the inclusion of information on obesity duration. Unfortunately, information on obesity duration was not available in the present study.

There is ongoing debate on how to define the MH subgroup [8]. The risk for CVD and mortality is already increased with the presence of one component of the MetS [39]. In this context, the PK criteria, which define MH by the fulfilment of all included criteria may be more appropriate to determine “true” MH [30] than the ATPIII criteria, which define MH when at least three criteria are fulfilled. Among other factors, MH status is affected by body fat distribution [7]. The BMI is probably not the ideal measure of obesity, since it does not discriminate between fat and lean body mass [40]. Therefore persons may be misclassified when BMI is used to define body size. WC is correlated with visceral adipose tissue and, as suggested by Plourde and Karelis [30], might be more appropriate to define MH body size groups. However, also WC does not account for body fat distribution in general [40].

The major limitation of this study was that blood sampling was not generally conducted in a fasting state. Thus, we used HbA1c instead of serum glucose and different cut-offs among fasting and non-fasting participants for triglycerides. Nevertheless, the decrease in the prevalence of low risk triglycerides may be overestimated. However, previous analyses of our data have shown that the decline in triglyceride levels persisted after adjusting for age and fasting period [18]. An additional limitation is that the WC measurement instructions slightly changed between the two German national health surveys; hence we cannot exclude misclassification bias, with overestimation of abdominal obesity in GNHIES98 compared to DEGS1. The slight increase in MH among persons with pre-obesity might be partially due to this change, since no significant change in MH was found in a sensitivity analysis excluding WC from the ATPIII criteria. The DEGS1 sample comprises first-time participants and re-participants of GNHIES98, which might differ in several aspects. Thus, we used weighting factors that account for this issue as well as for the complex sampling design and non-response. However, bias caused by the DEGS1 study design cannot be ruled out completely.

## Conclusion

In summary, proportions of adults with MH by body size categories were largely stable over time, except for an increasing proportion of MH women without abdominal obesity. Thus, the proportion of MH among people with obesity in Germany did not change over a period of ten years. Proportions of adults with MH vary according to body size and the MH criteria applied.

## Supporting Information

S1 Table Proportion of metabolically healthy persons according to ATPIII criteria excluding waist circumference. (DOCX)

## Abbreviations

CVD: cardiovascular disease
HbA1c: glycated haemoglobin
HDL-C: high density lipoprotein-cholesterol
MH: metabolically healthy
MU: metabolically unhealthy
PK: Plourde and Karelis
WC: waist circumference
