# Supplementary material for: Metabolic Health in Relation to Body Size: Changes in Prevalence over Time between 1997-99 and 2008-11 in Germany
Source: PLoS One. 2016 Nov 23;11(11):e0167159. doi: 10.1371/journal.pone.0167159 (PMC5120858; doi:10.1371/journal.pone.0167159)
Supplement: S1 Table — (DOCX) [file pone.0167159.s001.docx]

# S1 Table. Proportion of metabolically healthy persons according to ATPIII criteria excluding waist circumference.

|  | Total | | | Men | | | Women | | |
| --- | --- | --- | --- | --- | --- | --- | --- | --- | --- |
|  | GNHIES98 N=6,565 | DEGS1 N=6,860 | P | GNHIES98 N=3,205 | DEGS1 N=3,298 | P | GNHIES98 N=3,360 | DEGS1 N=3,562 | P |
| Normal weight | 83.0 (81.2-84.6) | 82.9 (81.1-84.6) | .140 | 79.0 (76.2-81.6) | 80.2 (76.5-83.4) | .091 | 85.7 (83.3-87.7) | 84.8 (82.8-86.6) | .650 |
| Pre-obesity | 58.3 (56.2-60.4) | 58.7 (56.4-60.9) | .170 | 56.0 (53.1-58.9) | 55.3 (52.1-58.4) | .470 | 62.0 (59.0-65.0) | 63.7 (60.0-67.2) | .230 |
| Obesity | 35.1 (31.8-38.4) | 36.8 (33.7-40.1) | .220 | 27.6 (23.8-31.7) | 34.2 (29.4-39.4) | .014 | 41.2 (36.6-46.0) | 39.4 (35.3-43.6) | .790 |

Proportion (%) of metabolically healthy persons by body size categories and survey, National Health Interview and Examination Surveys for Adults in Germany 1997-99 (GNHIES98) and 2008-11 (DEGS1)

ATPIII criteria excluding waist circumference (two out of four criteria fulfilled): HbA1c< 5.7 % and no diagnosis of diabetes and no use of antidiabetic medications; blood pressure< 130/85 mmHg and no diagnosis of hypertension and no use of antihypertensive medication; fasting triglycerides< 1.7 mmol/l or non-fasting triglycerides< 2.1 mmol/l [29] and no diagnosis of dyslipidaemia and no use of lipid-lowering medication; HDL-C≥ 1.03 mmol/l (men) or 1.30 mmol/l (women)

Due to Bonferroni-Holm correction p values < 0.01 were considered statistically significant (bold)
